# Academic Life in Emergency Medicine Blog and Podcast Watch: Respiratory Emergencies

**DOI:** 10.7759/cureus.2812

**Published:** 2018-06-14

**Authors:** Alice A Min, Eric J Morley, Salim R Rezaie, Sean M Fox, Andrew Grock

**Affiliations:** 1 Emergency Medicine, University of Arizona College of Medicine, Tucson, USA; 2 Emergency Medicine, Stony Brook Medicine, New York, USA; 3 Emergency Medicine/internal Medicine, University of Texas, San Antonio, USA; 4 Emergency Medicine, Carolinas Medical Center, Charlotte, USA; 5 Emergency Medicine, University of California Los Angeles, Los Angeles, USA

**Keywords:** emergency medicine, free open access medical education, respiratory emergencies

## Abstract

The Academic Life in Emergency Medicine (ALiEM) Approved Instructional Resources (AIR) Series and Approved Instruction Resources Professional (AIR-Pro) Series were created in 2014 and 2015, respectively, to address the need for curation of online educational content as well as a nationally available curriculum that meets individualized interactive instruction criteria. These two programs identify high-quality educational blog and podcast content using an expert-based approach.

We summarize the accredited posts on respiratory emergencies that met our a priori determined quality criteria per evaluation by eight experienced faculty educators in emergency medicine.

## Introduction and background

There has been a rapid rise of educational content available through blogs and podcasts in emergency medicine (EM) [1]. However, identification of quality resources for educators and learners has only made preliminary progress [2-4]. In addition, the Accreditation Council for Graduate Medical Education in 2008 endorsed a decrease in synchronous conference experiences for EM residency programs by up to 20% in exchange for asynchronous learning – termed Individualized Interactive Instruction (III) [5]. These developments created a need for both online content quality assessment as well as a nationalized curriculum that met III criteria.

To address these needs, the Academic Life in Emergency Medicine (ALiEM) Approved Instructional Resources (AIR) Series and Approved Instructional Resources Professional (AIR-Pro) Series were created in 2014 and 2015, respectively [6-7]. Using an expert-based, crowd-sourced approach, these two programs identify high-quality educational blog and podcast content. For the ALiEM Blog and Podcast Watch series, summaries of these posts are written by the AIR and AIR-Pro Series’ editorial boards [8-9]. This installment from the series summarizes the highest scoring social media educational resources on respiratory emergencies.

## Review

Topic identification

The AIR Series is a continuously building curriculum based on the Council of Emergency Medicine Residency Directors (CORD) testing schedule [10].

Inclusion and exclusion criteria

A search of the 50 most frequently visited sites per the Social Media Index [11] was conducted for resources relevant to respiratory emergencies, published within the previous 12 months. The search, conducted in August 2016, included blog posts and podcasts written in English for scoring by our expert panel.

Scoring

Extracted posts were scored without blinding by eight reviewers from the AIR Editorial Board, which is comprised of EM core faculty from various United States medical institutions. The scoring instrument contains five measurement outcomes using seven-point Likert scales: Best Evidence in Emergency Medicine (BEEM) score, accuracy, educational utility, evidence-based, and references (Table 1) [12]. More detailed methods are described in the original description of the AIR Series [6-7]. Board members with any role in the production of a reviewed resource recused him/herself from grading that resource.

**Table 1:** Approved Instructional Resources scoring instrument for blog and podcast content with the maximum score being 35 points. BEEM: Best evidence in emergency medicine; EP: Emergency physician; EBM: Evidence-based medicine.

Tier 1: BEEM Rater Scale	Score	Tier 2: Content Accuracy	Score	Tier 3: Educational Utility	Score	Tier 4: Evidence Based Medicine	Score	Tier 5: Referenced	Score
Assuming that the results of this article are valid, how much does this article impact on EM clinical practice?		Do you have any concerns about the accuracy of the data presented or conclusions of this article?		Are there useful educational pearls in this article for senior residents?		Does this article reflect evidence based medicine (EBM)?		Are the authors and literature clearly cited?	
Useless information	1	Yes, many concerns from many inaccuracies	1	Not required knowledge for a competent EP	1	Not EBM based, only expert opinion	1	No	1
Not really interesting, not really new, changes nothing	2		2		2		2		2
Interesting and new, but doesn't change practice	3	Yes, a major concern about few inaccuracies	3	Yes, but there are only a few (1-2) educational pearls that will make the EP a better practitioner to know or multiple (>=3) educational pearls that are interesting or potentially useful, but rarely required or helpful for the daily practice of an EP.	3	Minimally EBM based	3		3
Interesting and new, has the potential to change practice	4		4		4		4	Yes, authors and general references are listed (but no in-line references)	4
New and important: this would probably change practice for some EPs	5	Minimal concerns over minor inaccuracies	5	Yes, there are several (>=3) educational pearls that will make the EP a better practitioner to know, or a few (1-2) every competent EP must know in their practice	5	Mostly EBM based	5		5
New and important: this would change practice for most EPs	6		6		6		6		6
This is a "must know" for EPs	7	No concerns over inaccuracies	7	Yes, there are multiple educational pearls that every competent EP must know in their practice	7	Yes exclusively EBM based	7	Yes, authors and in-line references are provided	7

Resources with a mean evaluator score of ≥ 30 points (out of a maximum of 35) are awarded the AIR label. Resources with a mean score of 27-29 and deemed accurate and educationally valuable by the reviewers are given the Honorable Mention (HM) label.

Results

We initially included a total of 101 blog posts and podcasts. Key educational pearls from the four AIR posts and the eight Honorable Mentions are described.

Article 1. Swaminathan, A. Hemodynamically Unstable Pulmonary Embolism. (July 20, 2016) AIR

http://coreem.net/core/hd-unstable-pe/

This blog post reviews the pathophysiology, diagnosis, and management of massive pulmonary embolism.

Take Home Points:

Massive pulmonary embolism (PE), defined as a PE that results in hemodynamic compromise, is based on the patient’s physiologic response to the PE, not clot size. Intubation techniques should address massive PE’s complex physiologic derangements – baseline hypoxia, hypotension, and acidemia. Intravenous fluids should be administered cautiously to avoid right ventricular (RV) distention and left ventricular (LV) compromise. Ketamine or etomidate at reduced dosages is ideal agent for induction. Advanced diagnostic imaging is often not possible due to hemodynamic instability. Bedside assessment using point of care ultrasound along with electrocardiogram (EKG) or chest X-ray can provide information with which to make the diagnosis.

Systemic thrombolytics are indicated in massive PE with a mortality improvement (number needed to treat [NNT] = 16), but can also cause major bleeding (number needed to harm [NNH] = 10). The dose of alteplase is 100 mg intravenously over two hours. Surgical thrombectomy or intra-arterial thrombolytics are reasonable alternatives [13].

Article 2. Swaminathan, A. Life-Threatening Asthma. (September 30, 2015) AIR

http://coreem.net/core/life-threatening-asthma/

This post describes the management of life-threatening asthma exacerbations.

Take Home Points:

The immediate management goals are:

1. Stave off intubation while medications are given time to take effect.

2. Maximize pre-intubation parameters including pre-oxygenation and intravascular volume just in case.

3. Reverse bronchoconstriction to prevent respiratory failure from respiratory muscle exhaustion and decrease work of breathing.

Hypoxia may not be evident until late in the presentation. High-flow nasal cannula can provide increased oxygen flow compared to the standard nasal cannula. Non-invasive positive pressure ventilation (NIPPV) may also be beneficial to decrease work of breathing and improve gas exchange.

Intramuscular or intravenous epinephrine can be life-saving! Other options include magnesium 2 gm intravenously over 15 minutes and heliox, though this has not been well studied in the sickest asthmatics.

Intubation for severe asthma exacerbation does not fix the underlying problem – albuterol, epinephrine, and other bronchospasm medicines must be continued. Intubation can be dangerous in asthmatics due to hyperinflation and rapid acidosis if respirations are not matched during intubation or post-intubation. A strategy of permissive hypercapnia may help to avoid hyperinflation. Use ketamine for delayed sequence intubation if needed for NIPPV to aid in pre-oxygenation.

Recommended initial ventilator settings are: respiratory rate six to ten breaths per minute, tidal volume of six to eight milliliters/kilogram (ideal body weight), positive end-expiratory pressure of zero to five centimeters of water, fraction of inspired oxygen (FiO2) necessary to maintain oxygen saturation greater than 93%, and inspiratory flow rate of 100-120 liters/minute.

The DOPES mnemonic for troubleshooting instability on the ventilator is:

D = displacement of the endotracheal tube

O = obstruction of the endotracheal tube

P = pneumothorax

E = equipment failure

S = stacked breaths

Article 3. Curry, W. Status Asthmaticus, “Answers”. (August 26, 2015) AIR

https://emlyceum.com/2015/08/26/status-asthmaticus-answers/

This blog post discusses four key treatments of status asthmaticus: NIPPV, inhaled corticosteroids for discharged patients, ketamine for delayed sequence intubation, and epinephrine use.

Take Home Points:

NIPPV appears to benefit hospital length of stay and respiratory function parameters in patients with severe asthma. It does not appear to improve mortality or need for intubation however.

For persistent asthma patients discharged from the emergency department (ED), initiating outpatient inhaled corticosteroids should be strongly considered.

Ketamine may provide bronchodilation and seems to improve pre-intubation hypoxia in delayed sequence intubation.

Severe bronchoconstriction may limit drug delivery for inhaled drugs. As such, intravenous or intramuscular epinephrine may result in better drug delivery in severe asthmatics.

Article 4. Helman, A. Management of Acute Pediatric Asthma Exacerbations. (April 12, 2016) AIR

https://emergencymedicinecases.com/pediatric-asthma/

This post provides a thorough review of pediatric asthma management.

Take Home Points:

The Preschool Respiratory Assessment Measure (PRAM) and Pediatric Asthma Severity Score (PASS) scores can help identify severe asthma. Markers for a severe exacerbation include: history of previous life-threatening asthma exacerbations, intubation, ICU admission, worsening while on steroids, frequency of beta-agonist use, cardiopulmonary or psychiatric comorbidities.

Venous blood gases rarely help bedside management, though they may serve as a baseline for inpatient care.

Consider chest X-ray in those with fever, focal chest findings, subcutaneous emphysema, history of choking, or first-time wheezers.

Albuterol efficacy is identical between nebulized (2.5 mg) and metered-dose inhaler with spacer-delivered (4 puffs). Nebulized albuterol may be easier to administer to the very sick and young infants. Monitor for hypokalemia when giving repeated doses of albuterol.

Up to three doses of ipratropium bromide should be given as well. Patients and parents may find single-dose dexamethasone easier than a multiple-day course of oral prednisolone. Maximum doses of steroids should not exceed fluticasone 100 mcg twice per day or beclamethasone 100 mcg four times per day to avoid adrenal shock.

If not significantly improved after 60 minutes of treatments, intravenous magnesium sulfate may be helpful. Consider a fluid bolus prior to magnesium to prevent hypotension.

The benefit of epinephrine, ketamine, high flow oxygen, bipap, and heliox is equivocal.

Article 5. Goodnough, R. Bi-Level Ventilation: Who Needs It and Who Doesn’t? Pearls and Pitfalls. (August 19th 2016) HM

http://www.emdocs.net/bi-level-ventilation-needs-doesnt-pearls-pitfalls/

This blog post provides a basic overview of NIPPV, the indications for NIPPV, and some pitfalls.

Take Home Points:

NIPPV may help prevent avoidable intubations and its resulting complications. While continuous positive airway pressure (CPAP) provides continuous airway pressure to help recruit alveoli, decrease the work of breathing, and improve oxygenation, bilevel positive airway pressure (BiPAP) does all this and provides ventilatory support.

Indications:

1. Chronic obstructive pulmonary disease (COPD): BiPAP in COPD has a mortality benefit (NNT = 10) and decreased intubation rate (NNT = 4). For COPD exacerbations, use BiPAP for: (1) pH < 7.35 or partial pressure of arterial carbon dioxide (PaCO2) > 45 millimeters of mercury (mmHg) (2) severe dyspnea with signs of increased work of breathing or (3) severe respiratory acidosis (though failure rates may be as high as 50% when the patient’s pH is <7.25).

2. Cardiogenic pulmonary edema (CPO): Most NIPPV studies in CPO are for CPAP and not BiPAP. CPAP alone has been shown to decrease intubation rates and in-hospital mortality. Limited evidence suggests BiPAP may be associated with more rapid improvement in pH, PaCO2, partial pressure of arterial oxygen (PaO2), heart rate, work of breathing, afterload, preload, cardiac index and ejection fraction. However, it is unclear if either modality is more beneficial in regard to intubation rates or mortality. Indications for NIPPV in CPO include (1) respiratory failure, (2) hypercapnia and (3) increased work of breathing.

3. Asthma: Limited data and unclear benefit for NIPPV in asthma. The post recommends using NIPPV very carefully in this population and in conjunction with standard medical therapy.

4. Blunt thoracic trauma: Decreases intubation rates for patients with significant blunt thoracic trauma.

5. Palliative care: Consider in patients at end of life when intubation is not a viable option.

NIPPV Pitfalls:

(1) Can cause hypotension.

(2) Avoid if significant facial trauma or heavy oral secretions.

(3) Intubate if needed without delay.

(4) Awake patients only.

(5) Watch closely if the patient is severely acidotic.

(6) Monitor for auto-positive end-expiratory pressure (PEEP).

Article 6. Mallin, M and Dawson, M. Lung Ultrasound Basics Part 1: Pneumothorax and Pulmonary Edema. (2016) HM

http://www.ultrasoundpodcast.com/2016/05/lung-ultrasound-basics-part-1-pneumothorax-pulmonary-edema-ultrasoundmd-foamed-also-www-cabofest2017-com/

This post focuses on how bedside ultrasound (US) can narrow the differential or even identify the definitive diagnosis in undifferentiated shortness of breath (SOB).

Take Home Points:

Physical exam and history can be unreliable for differentiating COPD exacerbations and pulmonary edema. Bedside US has a positive likelihood ratio (LR+) of 7.4-12 and a negative likelihood ratio (LR-) of 0.06-0.16 for diagnosing pulmonary edema – easily outperforming history and physical exam.

Of note, bedside US performed after pulmonary edema treatments may not find the typical B lines and this changes the operating characteristics of US.

Bedside US performs excellently in the diagnosis of pneumothorax with a sensitivity of 86-98%. In contrast, auscultation and supine X-ray (which typically takes 6-10 minutes longer to perform than US) have a sensitivity of 50-62% and 28-75%, respectively.

Article 7. Rezaie, S. Is Apneic Oxygenation Overhyped with Scott Weingart. (April 4, 2016) HM

http://rebelem.com/is-apneic-oxygenation-overhyped-with-scott-weingart/

This blog post and podcast reviews the preoxygenation, denitrogenation, and apneic oxygenation during rapid sequence intubation (RSI).

Take Home Points:

The first preoxygenation step breaks down into two separate terms: preoxygenation and denitrogenation. Preoxygenation (PreOx) involves maximizing oxygen delivery prior to RSI medications. Denitrogenation is the process of replacing the lung’s nitrogen with oxygen. Apneic oxygenation (ApOx) is a passive movement of oxygen to the alveoli without spontaneous patient breaths or you providing the respirations. In patients with pulmonary shunt physiology, positive end-expiratory pressure (PEEP), also termed apneic CPAP, is a superior strategy as it provides both oxygen and improves alveolar recruitment. The bottom line of this post is essentially, PreOx and ApOx are all attempts to avoid deoxygenation while managing patients airways.

Article 8. Lubberdink A. TRanslating Emergency Knowledge for Kids (TREKK) Series | Bronchiolitis Guidelines (July 19, 2016) HM

https://canadiem.org/trekk-series-bronchiolitis-guidelines/

This blog post focuses on bronchiolitis diagnosis and treatment.

Take Home Points:

Bronchiolitis is typically caused by viral infection (most commonly respiratory syncytial virus) in the terminal bronchiolar epithelial cells leading to obstruction and bronchospasm of the distal airways. The diagnosis of bronchiolitis is a clinical one (i.e., based on history and physical exam). If you are confident in your diagnosis, additional testing, such as chest radiograph, blood cultures, and viral studies, is not helpful or indicated. A blood gas could be useful if there is a concern for potential respiratory failure. The cornerstone of treatment is supportive care such as adequate hydration and oxygenation (indicated if SaO2 < 90%), suctioning of nares, and antipyretics as needed. Therapies with equivocal evidence include: nebulized epinephrine, nebulized 3% hypertonic saline, and a combination of nebulized epinephrine and oral dexamethasone. Finally, the evidence does not support salbutamol, corticosteroids, or antibiotics.

Article 9. Guinney, A. Is Too Much Supplemental O2 Harmful in COPD Exacerbations? (Dec 3, 2015) HM

http://rebelem.com/is-too-much-supplemental-o2-harmful-in-copd-exacerbations/

This blog post reviews the myths of exogenous oxygen in COPD.

Take Home Points:

The three theories discussed are: oxygen-induced hypoventilation, the Haldane effect, and ventilation/perfusion mismatch.

Oxygen-induced hypoventilation: Hypercarbia and hypoxemia primarily drive the respiratory rate. In chronically hypercarbic COPD patients, oxygen level is believed to be the primary trigger for respiratory drive. However, evidence supporting this theory is conflicting. Supplemental oxygen was shown to have no effect on longer-term minute ventilation in one study while showing decreased minute ventilation in another.

Haldane effect: Deoxygenated hemoglobin better binds carbon dioxide (CO2) compared to oxygenated hemoglobin. However, supplemental oxygen shifts the equilibrium from deoxygenated to oxygenated hemoglobin which results in higher levels of carbon dioxide. While the chemistry here is sound, the patient-centered evidence demonstrates that the Haldane effect cannot account for all the hypercarbia observed – meaning something else must be involved.

Ventilation/perfusion mismatch: COPD patients with diseased lungs allocate perfusion to parts of the lungs with ventilation and away from the parts without. Administration of oxygen could alter this balance. In diseased sections of lung, this increased PaO2 could steal perfusion away from better functioning areas. In CO2 retaining patients, this theory makes sense, but it does not apply to non-CO2 retainers. Again conflicting evidence results in a murky picture as to the role of ventilation/perfusion mismatch in hypercarbia.

Article 10. Fox, S. Down Syndrome Airway (February 26, 2016) HM

http://pedemmorsels.com/down-syndrome-airway/

This blog post reviews the particular anatomy and physiology with Down Syndrome that affects airway management.

Take Home Points:

Not only are Down Syndrome patients more prone to respiratory illness secondary to a smaller and more narrow upper airways, smaller trachea, less functional cilia, and general hypotonia, but their specific physiologic features can complicate endotracheal intubation. Down Syndrome patients have less functional residual capacity and thus will be more prone to rapid desaturations. Their general hypotonia and relatively larger tongues result in more easy upper airway obstruction. Appropriate positioning is key in pre-oxygenation and airway alignment. Due to their smaller airways, a smaller endotracheal tube should be used as well. Lastly, atlantoaxial instability can lead to fractures with aggressive positioning. Techniques that minimize neck manipulation and maintain in-line stabilization may avoid this tragic complication.

Article 11. Boyd, A. Single Dose Dexamethasone (August 11, 2016) HM

http://rebelem.com/single-dose-dexamethasone-or-5-days-of-prednisone-in-adult-asthmatics/

This blog post reviews the current evidence for single dose dexamethasone for adult patients presenting with asthma exacerbation.

Take Home Points:

The recent randomized controlled, noninferiority trial by Rehrer et al. evaluated single dose of dexamethasone versus five days of prednisone [14]. It did not establish that dexamethasone was noninferior, as the dexamethasone group had an absolute increase in relapse or return visit of 2.3%. The authors and reviewers nonetheless conclude that the potential benefits of increased compliance with single dose regimens make it a reasonable alternative to five days of prednisone.

Article 12. Swaminathan, A. Basic Asthma Management (August 12, 2015) HM

http://coreem.net/core/basic-asthma-management/

This blog post reviews the basic management of asthma exacerbations.

Take Home Points:

Asthma exacerbations are diagnosed based on clinical presentation and examination. Diagnostic testing is not helpful unless alternative diagnoses are clinically suspected. The primary therapies for acute asthma exacerbations are inhaled beta-2 agonists, inhaled anticholinergics, and systemic corticosteroids. Additionally, intravenous magnesium has been shown to help decrease hospitalization rates in patients refractory to standard therapies.

## Conclusions

The ALiEM Blog and Podcast Watch series identifies high-quality educational blogs and podcasts for EM clinicians through its expert panel, using an objective scoring instrument. These social media resources are currently curated in the ALiEM AIR and AIR-Pro Series, originally created to address EM residency needs. While this article focuses on respiratory emergencies, additional AIR modules address other topics in emergency medicine. The resources chosen specifically for respiratory diseases are herein shared and summarized to help clinicians and educators filter through the rapidly published multitude of blog posts and podcasts. Our search was limited to content produced within the previous 12 months from the top 50 Social Media Index sites. While these lists are by no means a comprehensive analysis of the entire Internet for this topic, the AIR and AIR-Pro series provide post-publication accreditation and curation of recent online content to identify and recommend high-quality educational social media content for the EM clinician.
